# Recent Advances in the Preparation and Application of Two-Dimensional Nanomaterials

**DOI:** 10.3390/ma16175798

**Published:** 2023-08-24

**Authors:** Ying-Tong Guo, Sha-Sha Yi

**Affiliations:** College of Materials Science and Engineering, Zhengzhou University, Zhengzhou 450001, China; intenguo@163.com

**Keywords:** two-dimensional nanomaterials, chemical vapor deposition, graphene, catalysis

## Abstract

Two-dimensional nanomaterials (2D NMs), consisting of atoms or a near-atomic thickness with infinite transverse dimensions, possess unique structures, excellent physical properties, and tunable surface chemistry. They exhibit significant potential for development in the fields of sensing, renewable energy, and catalysis. This paper presents a comprehensive overview of the latest research findings on the preparation and application of 2D NMs. First, the article introduces the common synthesis methods of 2D NMs from both “top-down” and “bottom-up” perspectives, including mechanical exfoliation, ultrasonic-assisted liquid-phase exfoliation, ion intercalation, chemical vapor deposition, and hydrothermal techniques. In terms of the applications of 2D NMs, this study focuses on their potential in gas sensing, lithium-ion batteries, photodetection, electromagnetic wave absorption, photocatalysis, and electrocatalysis. Additionally, based on existing research, the article looks forward to the future development trends and possible challenges of 2D NMs. The significance of this work lies in its systematic summary of the recent advancements in the preparation methods and applications of 2D NMs.

## 1. Introduction

Since Geim’s successful preparation of single-layer graphene from graphite in 2004 [1], this material has attracted widespread attention due to its exceptional physical and electrochemical properties, including being a two-dimensional (2D) carbon material with excellent gas adsorption, high strength, ultra-low weight, low resistivity, and a room temperature Hall effect [2,3,4,5,6]. Despite its wide applications in sensors, biomaterials, energy, and other fields [7,8,9], graphene still lacks semiconductor properties due to its zero band gap. Furthermore, its high production cost and limited scalability restrict its application exclusively to high-end industries [10].

Up to now, there exists a wide range of 2D nanomaterials (NMs), including transition metal dichalcogenides (TMDs, such as MoS_2_, WSe_2_, and PtSe_2_), hexanol boron nitride (h-BN), graphene, noble metal dichalcogenides (NMDs like PdSe_2_, PtSe_2_, and PtS_2_), elemental 2D materials (such as stannene, borene, and black phosphorus), layered double hydroxides (LDHs), graphite carbon nitrides (g-C_3_N_4_), MXene, and so on [11,12,13,14,15,16,17,18]. These materials have a layered structure, and the mobility of electrons is limited to nanometer lengths, which is comparable to being several atomic layers thick [19], exhibiting properties similar to those of graphene. When compared to three-dimensional (3D) and one-dimensional (1D) NMs, 2D NMs have the following advantages: (1) a controllable thickness, which can be precisely controlled through various preparation methods, typically ranging from a few nanometers to tens of nanometers [20]. (2) A special electronic structure, including a regulated band gap, a distinctive Fermi surface, and a band structure [21]. (3) High specific surface area, which enhances their adsorption capacity and reactivity [22]. (4) Unique optical and electrical properties, including exceptional light absorption, fluorescence, and Raman scattering [23]. (5) Controllable physical and chemical properties, such as band gap, electrical conductivity, electrochemical activity, etc. The advantages of 2D NMs make them highly promising for a wide range of applications, including adsorption, catalysis, sensing, electronics, optoelectronics, and other fields [24]. TMDs exhibit robust intralayer covalent bonding and weak interlayer van der Waals interactions, exceptional electrical conductivity, and a large surface area, making them ideal candidates for use as counter electrodes (CEs) in dye-sensitized solar cell (DSSC) devices. In DSSCs, TMD-based electrocatalysts play a crucial role in facilitating redox reactions. It has been observed that maintaining a consistent supply of electrons on the surface of TMDs can greatly enhance their electrochemical activity [25].

A major challenge in the current development of 2D NMs lies in their synthesis and preparation. Two-dimensional NMs can be synthesized by using physical and chemical methods, including mechanical exfoliation, ion intercalation, ultrasonic-assisted liquid phase exfoliation, chemical vapor deposition (CVD), and hydrothermal synthesis [26,27,28,29]. Moreover, the 2D NMs exhibit different properties depending on their preparation conditions [30]. It is highly significant to investigate the cost-effective and efficient preparation of 2D NMs and to enhance their functionality in the relevant application fields.

Herein, this paper provides an overview of the preparation methods for 2D NMs and discusses the advantages and limitations of each method. Additionally, this article provides a summary of the applications of 2D NMs in various fields, such as sensors, lithium-ion batteries, photodetectors, electromagnetic wave absorption, and photocatalysis. The existing challenges for the future development of 2D NMs are also discussed. We anticipate that this review will further facilitate the design and preparation of high-quality and high-yield 2D NMs, thereby expanding their applications in sensor technology, electrochemistry, photocatalysis, and electrocatalysis.

## 2. Primary Morphology Parameters of 2D NMs

The primary physical properties of 2D NMs encompass electrical, optical, and mechanical properties. These properties can be influenced by altering the physical parameters, such as size, thickness, and surface defects [31].

### 2.1. Size

The physical properties of 2D NMs can be effectively fine-tuned by controlling their size and morphology. The electronic properties of 2D NMs exhibit quantum confinement effects when their size approaches the characteristic length scale of the electrons, resulting in discrete energy levels and bandgap modulation. As the size decreases, there is an increase in the bandgap, leading to semiconducting behavior. The tunability of electronic properties according to size provides opportunities for tailoring the conductivity and band structure of 2D NMs for specific applications [32]. The optical properties of 2D NMs, including absorption, emission, and light-matter interactions, can be effectively modulated by manipulating their sizes. The size-dependent energy band structure significantly influences the absorption and emission spectra, enabling precise control over the wavelength range at which the material absorbs or emits light. Additionally, quantum confinement effects can result in size-dependent photoluminescence, wherein the emission wavelengths shift towards higher energies as the size decreases [33]. The chemical reactivity of 2D NMs can be modulated by variations in size. As the size decreases, the proportion of edge atoms to bulk atoms increases, resulting in a higher density of reactive sites. This increased surface-to-volume ratio can enhance the material’s reactivity toward chemical species, making them suitable for catalytic applications [34].

### 2.2. Thickness

The thickness of 2D NMs significantly influences their electrical, optical, and mechanical properties. In terms of electrical properties, the thickness determines the electronic band structure and electron transport characteristics of a 2D material. As the thickness of 2D NMs decreases, the band structure changes, resulting in a variation in the electron’s band gap. Typically, thin 2D NMs have larger band gaps, resulting in better electron transport characteristics and enhanced carrier mobility [35]. Regarding optical properties, the thickness of 2D NMs affects their optical absorption, transmission, and reflection characteristics. Because thin 2D NMs absorb incident light more efficiently, they generally demonstrate higher optical absorption rates, improving higher photoelectric conversion efficiency and enabling a faster light response [36]. The thickness of the 2D NMs also affects its mechanical properties. Thin 2D NMs are more flexible and bendable, making them better adapt to different surface topographies and strain conditions. In summary, the electrical, optical, and mechanical properties of 2D NMs can be precisely controlled by adjusting their thickness, thus optimizing their performances [37].

### 2.3. Surface Defects

The surface defects in 2D NMs have significant effects on their physical properties. The presence of surface defects can cause electron scattering and alter the band structure, introducing energy levels that affect carrier transport and conductivity. Furthermore, surface defects can lead to the localization of charges and a decrease in electron lifetime, thereby influencing the material’s electron transport capabilities [38]. Regarding optical properties, surface defects can alter the material’s optical absorption, refractive index, and transmittance. These alterations can affect optical properties, such as luminescence characteristics and optical transparency [39]. In summary, the presence of surface defects significantly affects the electrical, optical, and mechanical properties of 2D NMs. Consequently, it is imperative that surface defects be considered and controlled in the design and application of 2D NMs.

## 3. Preparation of 2D NMs

The preparation of 2D NMs is of great significance for related research, with varying requirements for the surface morphology, lateral dimensions, and microstructure, depending on the intended application field. The preparation techniques for 2D NMs have expanded with the progress of research and can now be broadly classified into “top-down” and “bottom-up” approaches [40]. Different materials or the same material produced using various preparation techniques will have certain differences in their properties.

### 3.1. Top-Down Method

The top-down preparation method involves the gradual removal of matrix material under an external force. The top-down approach relies on exfoliating thin layers of 2D crystals from their parent layered bulk crystals. It should be noted that top-down methods are only applicable to materials with layered compound bulk crystals. In this review, our focus has been on mechanical peeling, ultrasonic-assisted liquid phase exfoliation, and ion intercalation-assisted exfoliation [41].

#### 3.1.1. Mechanical Peeling

**Figure 1:**
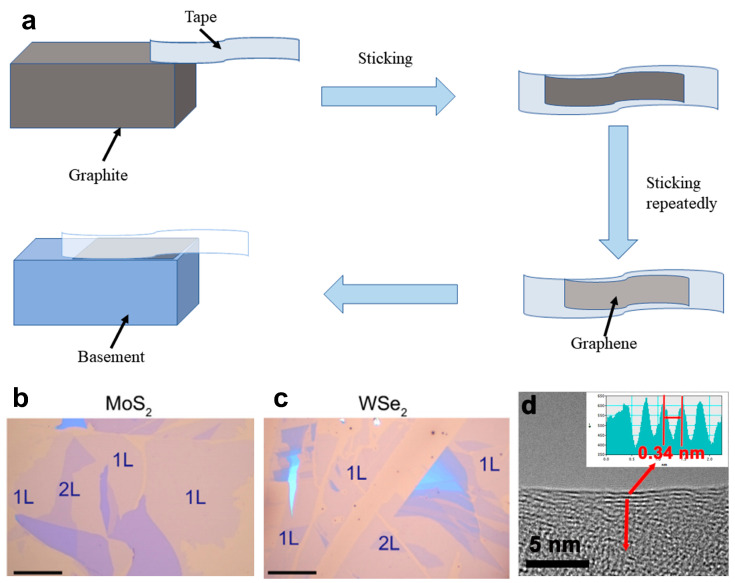
(**a**) Mechanism diagram of mechanical peeling method. Optical images of large-scale 2D materials on polycrystalline Au obtained by the UHV mechanical exfoliation technique: (**b**) MoS_2_ and (**c**) WSe_2_. The 1L and 2L areas are indicated in each picture. Scale bar: 40 μm [44]. Copyright 2022, Science Bulletin. (**d**) The HRTEM image of a single Pebax-BNNS with five layers. Inset is the corresponding distance profile of the area marked with the red line in d, showing a crystal spacing of 0.34 nm [45]. Copyright 2019, NPJ 2D Materials and Applications.

As early as 2004, Geim and his colleague Novoselov adopted the technique of mechanical exfoliation (repeated peeling) [1] to fabricate high-quality graphene films with a thickness of only a few atomic layers (Figure 1a). The mechanical peeling method applies external force to the matrix material through special adhesive tapes, ball milling, and other means. As a result, the matrix material is pulverized to obtain 2D NMs [42]. This method ensures high sample purity and is cost-effective because it does not involve chemical reagents or chemical reactions in the preparation process. It does, however, also have several drawbacks, including low yield, poor controllability, and the impossibility of large-area and large-scale preparation.

Huang et al. [43] proposed a novel method for the preparation of graphene. In the preparation process, aluminum (Al) is utilized as a grinding aid, and the Al particle diameter ratio is 200 mesh:500 mesh = 1:1.884. The result showed that the prepared graphene was of high quality with a specific surface area of 542.6 m^2^ g^−1^; the number of layers was predominantly less than five, and the yield was greater than 90%. However, the detergent utilized for Al removal may potentially cause damage to the graphene sample. To overcome this drawback, Sun et al. [44] utilized polycrystalline metal thin films containing Ag, Au, Fe, Cr, and other metals as the substrate. After pressing the crystal and substrate to ultra-high vacuum (UHV) conditions for several minutes, 2D NMs, such as graphene, FeSe, phosphorene, and MoS_2_, were successfully prepared, as shown in Figure 1b,c. This method has a wide range of applications. The treatment of precursors is a crucial aspect in the preparation of 2D NMs. An et al. [45] utilized Pabex as the precursor, which was preheated at 200 °C before adding BN solid powder for exfoliation. The microstructure of the as-obtained products was further characterized by high-resolution transmission electron microscopy (HRTEM) imagery, as shown in Figure 1d. The resulting preparation process produced functionalized BN nanosheets (NSs) without solvents and with simple procedures. The sample exhibits excellent water dispersibility and maintains the lamellar structure well under rubbing conditions. Deng et al. [46] synthesized a few layered MoS_2_ flakes with wrinkles through micromechanical exfoliation, revealing that the carrier mobility measured in the wrinkled part at 30 K (μ_w_ = 5.55 cm^2^ V^−1^ s^−1^) is much higher than that in the flat region (μ_f_ = 1.42 cm^2^ V^−1^ s^−1^). The increase in carrier mobility is attributed to the suppression of electron-phonon coupling and the reduction in lattice scattering caused by tensile strain-induced wrinkles in MoS_2_. Carrier mobility plays a crucial role in determining the operating frequency of electronic devices. For example, in bipolar transistors, the time it takes for a small number of carriers to cross the base region is a critical limitation on frequency response characteristics. A high carrier mobility can effectively reduce this transition time and enhance device performance. In some cases, strain can significantly increase the carrier mobility of 2D NMs.

#### 3.1.2. Ultrasonic-Assisted Liquid Phase Exfoliation

**Figure 2:**
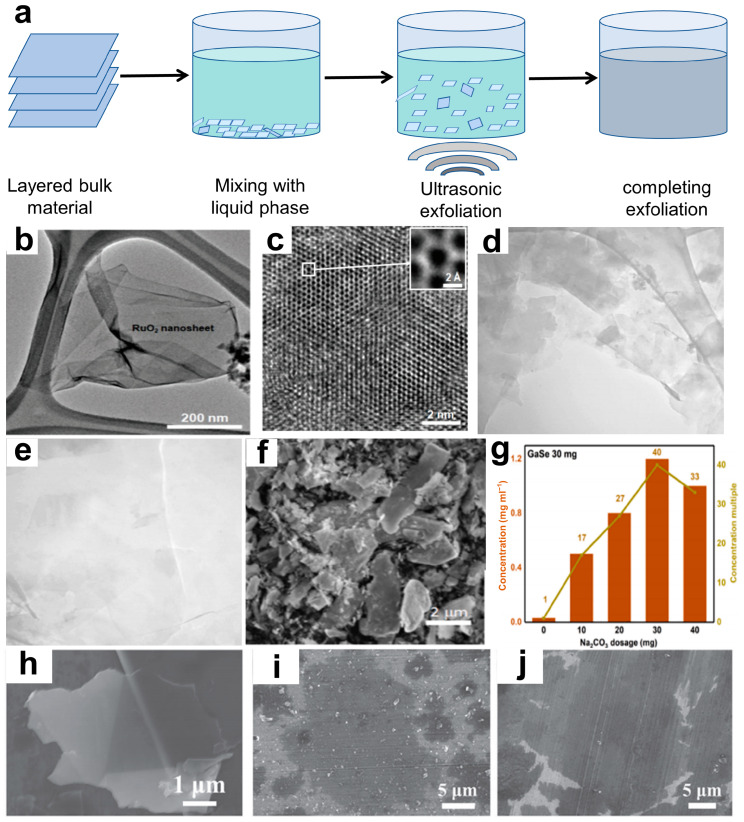
(**a**) Mechanism diagram of UALPE. (**b**) TEM image and (**c**) monochromated Cs-corrected HRTEM image of the exfoliated RuO_2_ nanosheet using 60 min of ultrasonication [50]. Copyright 2021, Inorganic Chemistry Frontiers. (**d**) TEM micrographs of the exfoliated graphene fakes, scale bar: 200 nm and (**e**) 100 nm [51]. Copyright 2022, Scientific Reports. (**f**) SEM image of the sample (the dosage of Na_2_CO_3_ is 30 mg). (**g**) The concentrations of different samples and the corresponding multiple-to-blank control group concentrations [52]. Copyright 2021, Materials Letters. SEM images of the obtained (**h**) graphene, (**i**) MoS_2_, and (**j**) WS_2_ nanosheets, respectively [53]. Copyright 2021, Small.

Ultrasonic-assisted liquid phase exfoliation (UALPE) is a process that utilizes ultrasonic waves in a suitable solvent to assist in the separation of bulk materials from top to bottom. Figure 2a illustrates the corresponding mechanism, which overcomes the van der Waals forces between material layers [47]. The selected solvent is crucial in determining the surface morphology and stability of 2D NMs during this process, as it must match the surface tension of the stripped material.

The primary function of the liquid phase in the stripping process is to act as a medium for efficient energy transfer, thereby facilitating the peeling off of materials under shear force influence. Additionally, the liquid phase plays a crucial role in lubrication and cleaning processes, enabling the production of high-quality 2D NMs. It is important to note that different types of liquid phases exhibit varying peeling efficiencies, necessitating careful selection based on specific target materials. For instance, when preparing GaSe, it is recommended to utilize isopropanol acid (IPA) as the liquid phase [48]. This technology was utilized to mix an aqueous surfactant solution with graphite, followed by exfoliation under ultrasonic-assisted conditions [49]. The resulting graphene-like materials not only possess a similar lamellar structure as graphene but also exhibit superior electrochemical performance and high flexibility. Kim et al. [50] conducted ultrasonic treatment on a RuO_2_ ion mixture after 3 days of an ion exchange reaction, resulting in a 50% increase in the yield of RuO_2_ NSs. Furthermore, a prolonged ultrasonic time led to a decrease in the lateral size of RuO_2_ NS decreases and reduced the energy required for peeling (Figure 2b,c). Besides, Mushfq et al. [51] investigated the impact of ultrasonic time and power on the quality and yield of fewer layered graphene samples (one–three layers). The findings indicate that the optimal sample quality and yield are achieved by ultrasonication for 55 min at a power output of 264 W. The TEM images demonstrate the presence of exfoliated multilayer (<10 layers) and fewer layered (one–three layers) samples (Figure 2d), with better quality and smaller-sized large graphene flakes (Figure 2e). In terms of the high-yield preparation methods, Qi et al. [52] proposed a feasible and effective approach for synthesizing GaSe NSs by adding Na_2_CO_3_ powder to IPA, which was demonstrated to increase the exfoliation yield of GaSe NSs by 40 times (Figure 2f,g). This method features simplicity, low costs, and convenient operation while also holding the potential for peeling other 2D NMs. Shi et al. [53] have developed a novel ultrasonic-ball milling strategy that effectively produces abundant high-quality ultrathin 2D NMs with a large transversal size by adding moderate Al_2_O_3_ abrasives to the precursor solution during ultrasound liquid-phase processing. The flake graphite, MoS_2_, and WS_2_ NSs obtained through this method have a lateral size ranging from 1 to 20 µm and a thickness of 1–3 nm, with a yield exceeding 20% (Figure 2h–j). This demonstrates the universality of this strategy in preparing highly representative nanomaterials.

#### 3.1.3. Ion Intercalation-Assisted Exfoliation

**Figure 3:**
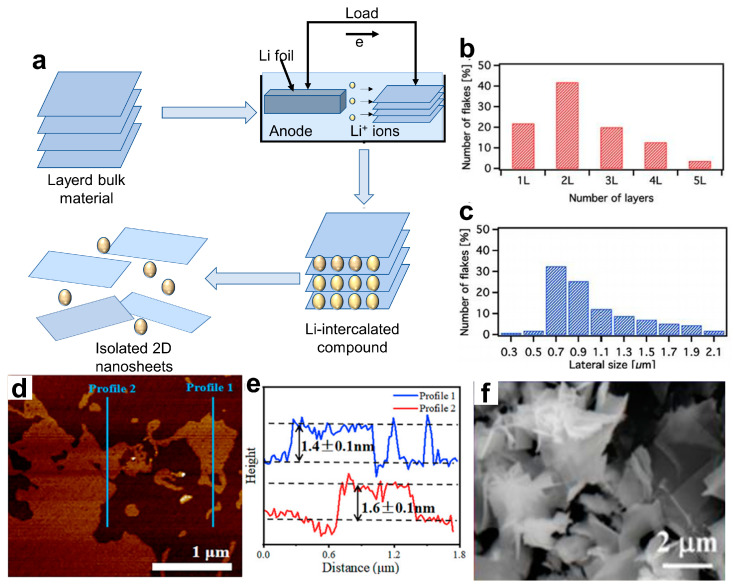
(**a**) Mechanism diagram of ion intercalation-assisted exfoliation method. (**b**) Thickness distribution obtained from scanning transmission electron microscopy (STEM) measurements on 60 different nanoflakes. (**c**) Lateral-size distribution based on data from 150 different nanoflakes [55]. Copyright 2018, Flat Chem. (**d**) AFM image and (**e**) corresponding height profiles of Ti_3_C_2_T_x_ [56]. Copyright 2022, IScience. (**f**) SEM image exfoliated WS_2_ nanosheets. Inset: Energy dispersive X-ray(EDX) element analysis results of corresponding positions in (**f**) [57]. Copyright 2021, Crystal Research and Technology.

In order to achieve the controlled synthesis of high-quality, large size 2D NMs, an ion intercalation-assisted stripping strategy has emerged. It involves the insertion of cations with a smaller ionic radius (such as lithium) into crystal gaps to form intercalation compounds, which can generate new vacancies through the complete filling and phase transition of lithium ions (Li^+^), resulting in layered materials [54]. The mechanism diagram for this method is shown in Figure 3a. Although this method can achieve high yields of 2D material NSs, it remains difficult to precisely control the amount of ion intercalation.

El Garah et al. [55] presented a fast (<1 h) exfoliation of MoS_2_ via lithium-ion intercalation by using a solution of lithium chloride (LiCl) in dimethyl sulfoxide (DMSO). The expansion and intercalation process of MoS_2_ bulk crystal is achieved over a time of 45 min by using a 1 M solution of LiCl in DMSO as the electrolyte and source of Li^+^. The sample is suitable for application in low-cost (opto-)electronic devices. The size and thickness of the resulting NSs were estimated by conducting statistical studies using scanning transmission electron microscopy (STEM) and HRTEM (FEI Tecnai F20 TEM equipped with a Schottky emitter) on 150 and 60 nanoflakes, respectively, obtained from different batches. The analysis revealed a significant presence of mono-, bi-, and tri-layer thick MoS_2_ nanoflakes with an average lateral size of ~0.8 μm. Combining various processes is another approach to improving the yield. Xv et al. [56] synthesized ternary-cation-intercalated Ti_3_C_2_T*_x_* MXene NSs by using mixed fluoride salt wet etching and an alkalization process. The Li, K, and Na ions were successively inserted into multilayered Ti_3_C_2_T_x_ to enlarge the distance between the layers and selectively weaken the interaction forces, thus achieving structural control with angstrom-level precision and a high surface area (92.6 m^2^ g^−1^). Figure 3d,e show the atomic force microscope (AFM) image. The yield of generated dispersed Ti_3_C_2_T_x_ can increase from 45% to 62.9% when compared with an ordinary method by single Li^+^ intercalation. Besides, Tian et al. [57] prepared WS_2_ NSs by Li^+^ intercalation exfoliation. Their research indicates that this method not only enables the large-scale preparation of WS_2_ NSs but also results in a larger lateral size and a well-defined lattice structure with no chemical impurities remaining, as illustrated in Figure 3f. Besides, the insertion and de-insertion of Li^+^ have important applications in the phase transition of 2D MoS_2_. For example, Hou et al. [58] successfully synthesized trigonal MoS_2_ (1T-MoS_2_) by inserting ion atoms (at a concentration of 20%) into the structure of hexagonal MoS_2_ (2H-MoS_2_), and the transition MoS_2_ (1T’-MoS_2_) phase was achieved through the de-insertion of Li atoms.

### 3.2. Bottom-Up Method

The bottom-up approaches employed for the synthesis of ultrathin 2D NMs involve controlled chemical reactions of specific precursors under well-defined experimental conditions. In principle, this bottom-up method offers greater versatility, enabling the potential fabrication of various types of ultrathin 2D NMs. This review primarily focuses on chemical vapor deposition (CVD), the hydrothermal method [59], physical vapor deposition (PVD), and atomic layer deposition (ALD), which can all be used to produce large-sized 2D NMs with good microstructure.

#### 3.2.1. Vapor Deposition

(1) Chemical vapor deposition

**Figure 4:**
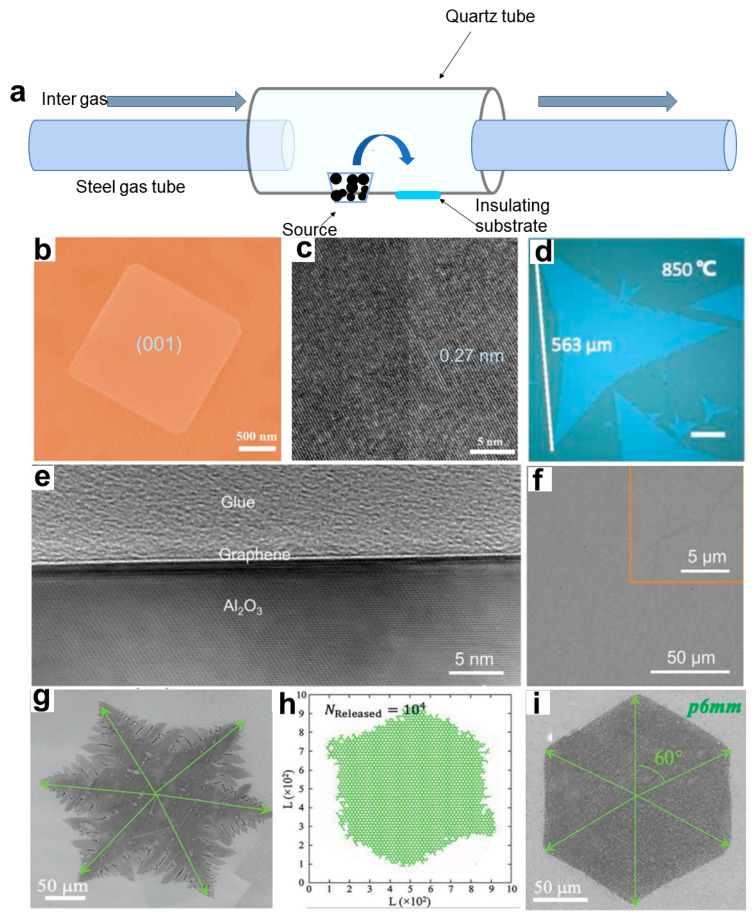
(**a**) CVD mechanism diagram. (**b**) SEM image of 2D BiOBr flakes. (**c**) HRTEM image of 2D BiOBr flakes [61]. Copyright 2020, Journal of Materials Science & Technology. (**d**) Optical image of large single-crystal monolayer MoS_2_. Scale bar, 100 μm [62]. Copyright 2018, Applied Physics Letters. (**e**) High-resolution cross-sectional TEM image of as-grown graphene on sapphire. (**f**) Typical SEM image of as-grown graphene on sapphire. The inset shows the high-magnification SEM image of graphene [63]. Copyright 2021, Science Advances. (**g**) Common-shaped graphene synthesized by CVD (scale bar: 50 µm). (**h**) A pattern of 2D-DLA simulation with the quasi-3D-release mode, where the defected holes can be noticed in the simulation result. (**i**) Perfect hexagonal graphene domain fabricated under a higher ratio of H_2_/CH_4_ (scale bar: 50 µm) [64]. Copyright 2019, Advance Materials.

CVD is a crucial technique in producing 2D NMs, where two or more solid raw materials are transformed into a gaseous state within a reaction chamber. The resulting gaseous reactants undergo chemical reactions to form volatile species, which are then transferred to the deposition area and deposited onto the substrate. This method is commonly used for producing 2D NMs with an extremely small thickness. Figure 4a depicts a mechanism diagram of the CVD process. Inert gases, such as nitrogen and argon, can be introduced to serve as auxiliary agents in order to reduce film wrinkles and increase flatness [60]. The advantages of this method lie in its superior control and avoidance of pollution during the preparation process when compared to the “top-down” method, as well as its wide range of applications. However, it is also subject to high costs, raw material volatility, and low reactant utilization rates.

Chen et al. [61] used BiBr_3_ powder and oxygen as the source to synthesize BiOBr NSs by a space-limited CVD method. Two mica substrates superimposed over each other were placed on a quartz boat in the center of the heating area. The space-confined CVD-grown BiOBr nanoflakes (Figure 4b,c) show extremely high crystalline quality and excellent UV photo-detecting performances. Zhang et al. [62] employed molten glass as the growth substrate for layered MoS_2_, resulting in larger samples with more regular edges. The maximum single crystal size of this sample reached 563 μm and exhibited superior electrical properties, as shown in Figure 4d. Chen et al. [63] utilized sapphire as a substrate for graphene growth, which was heated to 1400 °C and introduced with methane as the precursor. The resulting graphene film exhibited single-layer characteristics and high carrier mobility (14,700 cm^2^ V^−1^ s^−1^). Figure 4e presents a cross-sectional TEM image of the graphene/sapphire interface, demonstrating high uniformity without discernible contamination. In order to prepare graphene with higher regularity and fewer defects, Li et al. [64] built a 2D diffusion-limited aggregation (2D-DLA) model based on an atomic-scale growth mechanism by using a modified traditional fractal theory. The existence of a fractal-growth-based mechanism in the CVD synthesis of several 2D NMs was revealed. They first synthesized a sample of graphene by using CVD under the conditions of a growth environment of CH_4_ = 10 sccm, H_2_ = 10 sccm, and T = 1030 °C, which had some holes on its surface (Figure 4g). Then, they analyzed the 2D-DLA model and found out that when the release rate of the active carbon atoms was significantly decreased, the holes disappeared, and the shapes of the simulated patterns tended to be regular hexagons (Figure 4h). In light of these findings, they lowered the rate at which active carbon atoms were generated by increasing the ratio of H_2_/CH_4_ to 50 sccm: 5 sccm under the same growth temperature of 1030 °C; this allowed them to obtain a high-quality, defect-free, hexagonal graphene domain by using CVD growth (Figure 4i).

Metal-organic chemical vapor deposition (MOCVD) is a technology for epitaxial growth in the vapor phase, which has been developed based on the traditional method of vapor phase epitaxy (VPE). The MOCVD technique utilizes the organic compounds of group III and II elements, as well as the hydrides of the group V and VI elements, as source materials for crystal growth. By utilizing thermal decomposition reactions in VPE on a substrate, MOCVD can produce thin-layer single-crystal materials, including various III–V, II–VI compound semiconductors and their multilayer solid solutions [65]. Noh [66] utilized a metalorganic CVD system to synthesize rectangular-shaped 2D-layered Ge_4_Se_9_ using a liquid germanium precursor at 240 °C. Their MOCVD reactor is equipped with a two-zone heating system, allowing for the precise control of precursor decomposition in the first heating zone (T_1_ = 480 °C) and separate crystal synthesis in the second heating zone (T_2_ from 240 to 400 °C). In their experiment, Ge(dmamp)_2_ and dimethyl selenide (CH_3_)_2_Se were employed as the precursors for Ge and Se, respectively, while muscovite mica served as the substrate. In terms of research on core-shell NMs, Li et al. [67] reported Au@MoS_2_ by utilizing a modified CVD method to successfully grow fullerene-like MoS_2_ shells on Au nanoparticles, which were hetero-structured core-shell materials with great potential for future applications in optical imaging, sensors, and opto-electronics.

(2) Physical vapor deposition

**Figure 5:**
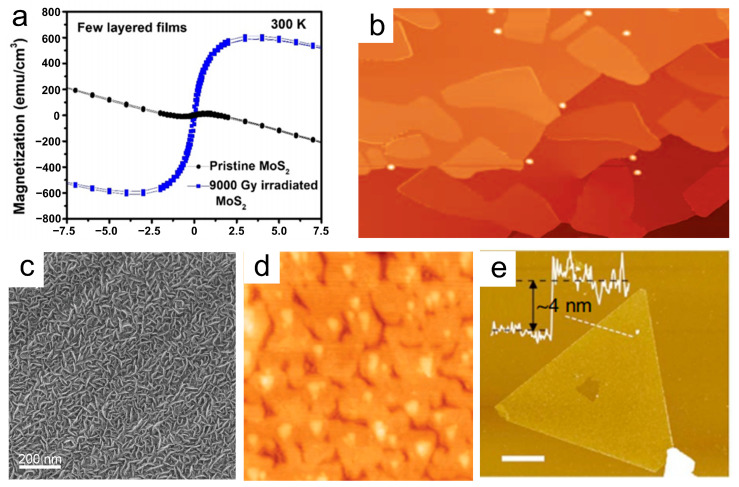
(**a**) Magnetization versus applied magnetic field for MoS_2_ films before and after 9 kGy irradiation measured at 300 K [69]. Copyright 2023, ACS Nano. (**b**) Large-scale STM topography image of borophene sheets. The scale bar is 100 nm [70]. Copyright 2015, Science. (**c**) SEM images of MoS_2_ nano-structured films were obtained under 100 W of sputtering power [71]. Copyright 2012, The Royal Society of Chemistry. (**d**) Zoomed-in STM image of stanene (40 nm × 40 nm) [72]. Copyright 2015, Nature Materials. (**e**) AFM image of a typical triangular antimonene sheet. The thicknesses are 4 nm. The scale bar is 1 μm [73]. Copyright 2016, Nature Communications.

Physical vapor deposition (PVD) is a widely employed technique for fabricating 2D NMs. In this process, a solid material is transformed into a vapor or ionic state, then deposited on a substrate surface to form a thin film. PVD offers several advantages, including high purity, uniform film deposition, and precise control. However, it also has some disadvantages, such as material selection limitations, the requirement for a high vacuum environment, and relatively slow growth rates [68]. Anbalagan et al. [69] investigated the impact of gamma-ray irradiation on MoS_2_ thin films by magnetron sputtering. They demonstrated in their experimental study that fewer-layered sputtered MoS_2_ films exhibit long-range ferromagnetic (FM) behavior with a magnetization (M_s_) of approximately 610 emu/cm^3^ at room temperature following 9 kGy of gamma-ray irradiation, as shown in Figure 5a. Molecular beam epitaxy (MBE) is an important PVD method. When utilizing this method, borophene and stanene can be successfully synthesized. Mannix et al. [70] first synthesized ultrathin borophene sheets under UHV conditions by utilizing a solid boron atomic source with a purity of 99.9999%. A scanning tunneling microscope (STM) image is shown in Figure 5b. This approach was chosen to avoid the challenges related to toxic precursors. The substrate temperature was maintained between 450 °C and 700 °C during the growth process, whereas the boron flux was maintained at approximately 0.01 to 0.1 monolayer per minute. Zhang et al. [71] fabricated MoS_2_ nanostructures onto carbon cloth by means of direct PVD using a magnetron sputtering system (Figure 5c). The results demonstrated that well-defined nanosheet arrays could be achieved with a sputtering power of 100 W at a gas pressure of 3 mtorr. Zhu et al. [72] developed ultrathin Sn films with 2D stanene structures on a Bi_2_Te_3_ substrate by using MBE. Antimonene is expected to possess potential applications in the field of flexible transparent conductive electrodes. Figure 5d shows a zoomed-in STM image of the stanene film. Ji et al. [73] achieved high-quality, fewer-layered antimonene polygons on a mica substrate by utilizing van der Waals epitaxy. As shown in Figure 5e, the antimonene polygons have a thickness of as low as 4 nm, which corresponds to approximately 10 atomic layers. This study lays the groundwork for further experimental investigations into the exceptional properties of antimonene and its potential applications.

#### 3.2.2. Hydrothermal Strategy

In the hydrothermal process, a specific precursor is immersed in an aqueous autoclave solution and subjected to high temperature and pressure conditions to initiate the hydrothermal reaction. The mechanism diagram is shown in Figure 5a. Afterward, it must undergo various post-treatment powder processing methods, including separation, washing, and drying procedures [74]. The hydrothermal method offers the benefits of facile operation, low costs, and a stable process, enabling the high-yield and high-quality production of 2D NMs. Moreover, it is possible to synthesize 2D NMs with various shapes and sizes by adjusting the reaction conditions [75].

**Figure 6:**
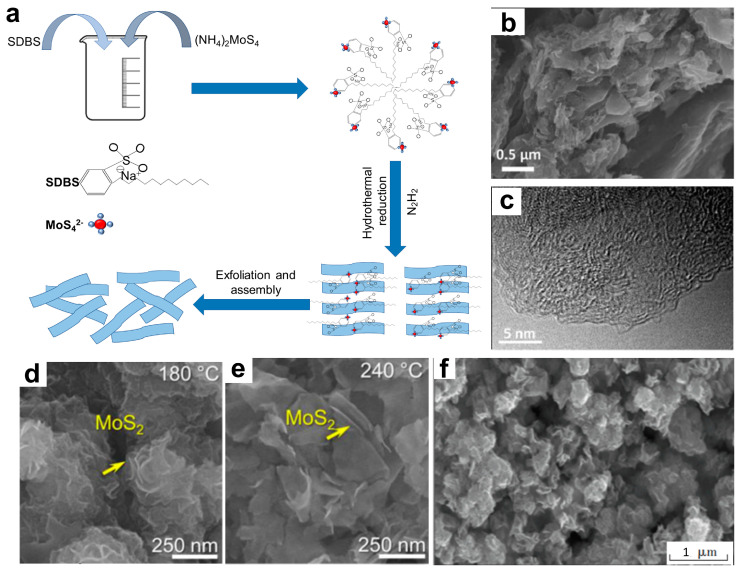
(**a**) Mechanism diagram of a hydrothermal method (taking the preparation of MoS_2_ assisted by sodium dodecyl benzene sulfonate (SDBS) as an example). (**b**) SEM and (**c**) TEM images of the 2D porous carbon sample [76]. Copyright 2017, The Royal Society of Chemistry. Field-emission SEM (FESEM) image of synthesized 2D MoS_2_ at (**d**) 180 °C and (**e**) 240 °C [77]. Copyright 2023, Journal of Sol-gel Science and Technology. (**f**) SEM image of MoS_2_ nanoparticles [78]. Copyright 2022, Inorganic Chemistry Communications.

Huang et al. [76] developed a new strategy to develop 2D morphology carbon via a facile one-pot hydrothermal method by using the biomolecule guanine and diverse carbohydrates as precursors. During the hydrothermal process, guanine drives the formation of the 2D nanostructures. The porous carbon obtained through this method exhibits thin layers, a high surface area, and nitrogen doping that can be adjusted (Figure 6b,c). As a result, the sample exhibits outstanding catalytic performance. Additionally, temperature plays a pivotal role in the fabrication of 2D MoS_2_ nanocomposites. Long et al. [77] successfully synthesized 2D MoS_2_ nanocomposites by using a simple hydrothermal method, resulting in ultrathin NSs with a thickness in the range of 6–13 nm (equivalent to six–eight layers) and an average lateral size of 130–330 nm. By increasing the hydrothermal temperature from 180 to 240 °C, MoS_2_ showed a preference for growth along the c-axis, and an improvement in crystal quality was observed (Figure 6d,e). Vidhya et al. [78] studied the effects of different hydrothermal synthesis temperatures on the morphology and properties of 2D MoS_2_. The experiments indicate that the samples prepared at a temperature of 160 °C exhibit exceptional electrical properties, as shown in Figure 6f. This electrode recorded a maximum specific capacitance of 691 F g^−1^ at a current density of 1 A g^−1^ and a good cyclic stability of 89% over 5000 cycles.

#### 3.2.3. Atomic Layer Deposition

**Figure 7:**
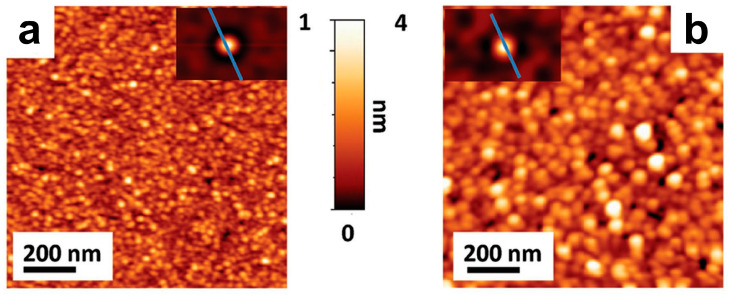
AFM topographic maps of (**a**) 4 nm thick ALD film precursor and (**b**) four MoS_2_ layers. The insets show the self-correlation functions calculated from the corresponding topographies [80]. Copyright 2016, Advanced Electronic Materials.

An atomic layer deposition (ALD) is a method for forming atomic layers by alternately introducing pulses of gas phase precursors into the reactor and undergoing chemical adsorption and reactions on the substrate. By supplying inert gases (Ar, N_2_, etc.) to different reactants, the individual reactants can be separated, enabling layer-by-layer deposition and repeated thickness control. In summary, the ALD technique provides precise control, uniformity, and versatility in the fabrication of 2D NMs. The method, however, does have its limitations, such as slower growth rates, equipment complexity, and restricted reaction conditions [79]. Martella [80] focused on the growth of a few layers of MoS_2_ through the sulfurization of molybdenum oxide precursor films deposited using the ALD technique, which uses Mo(CO)_6_ and ozone as the precursors. By leveraging the highly conformal growth capabilities of ALD, they were able to effectively manipulate the growth characteristics of the precursor film, ultimately impacting the quality of the resulting MoS_2_ layers. For 4 nm thick precursors, the growth of four MoS_2_ layers was obtained uniformly over a cm^2^ sample area. The AFM image is shown in Figure 7.

In summary, the top-down methods for the preparation of 2D NMs possess the advantages of cost-effectiveness, suitability for industrial production, and relatively simple preparation techniques, as well as some drawbacks, primarily the limited controllability, lower product quality, and limited applicability to specific materials. When compared to top-down methods, bottom-up methods offer superior controllability over the products and excellent quality, as well as the potential to synthesize virtually any material. There are, however, some drawbacks to this approach, including high costs and a relatively low yield [81] (Table 1).

## 4. Application of 2D NMs

Nanomaterials featuring ultrathin 2D structures exhibit distinctive physical, electronic, chemical, and optical properties, making them potentially valuable for a wide range of applications. Moreover, these diverse materials exhibit a broad range of compositions and properties, making them suitable for a variety of applications. Two-dimensional NMs have demonstrated significant potential in a multitude of fields, including electronics/opto-electronics, catalysis, energy storage and conversion, water purification, sensors, and biomedicine. In this section, we provide a comprehensive overview of the recent advances in the utilization of 2D NMs across various key applications, focusing specifically on their application as sensors, lithium-ion batteries, photodetectors, electromagnetic wave absorption, photocatalysis, and electrocatalysis [82].

### 4.1. Sensor

Sensors can be broadly categorized into gas sensors, biosensors, and other types. The gas sensor is primarily utilized for the detection of a specific gas. It works by converting information about the concentration of the gas through physical or chemical principles and subsequently outputting the corresponding electrical signals. Currently, the primary measurement parameters for gas sensors include sensor responsivity, response and recovery time, selectivity, and stability. The exceptional gas adsorption capacity, large specific surface area, excellent electrical conductivity, and stable chemical properties of 2D NMs, such as graphene and TMDs, make them highly promising for use in gas sensors [83].

**Figure 8:**
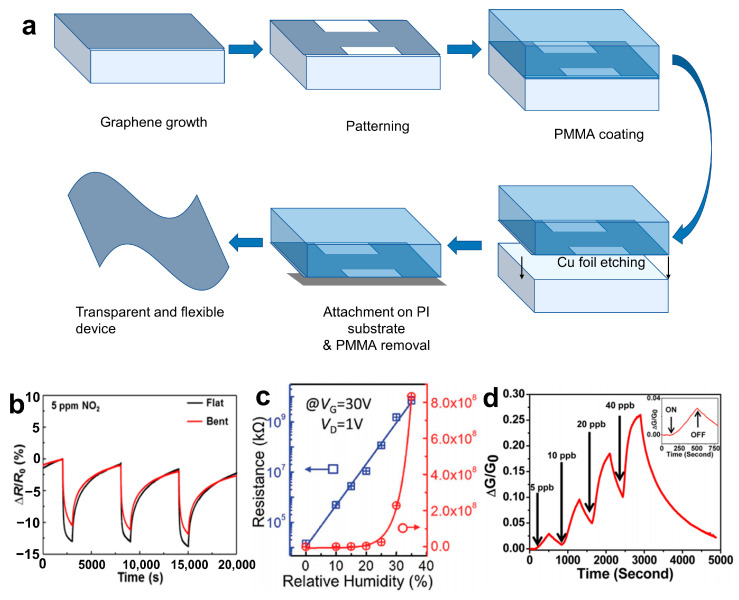
(**a**) Gas sensor preparation mechanism diagram. (**b**) Response curves of the sensor without and with the bending strain [84]. Copyright 2015, American Chemical Society. (**c**) The resistance variation at different RHs (VG = 30 V), the blue and red lines show increment trends in linear and logarithmic co-ordinates, respectively [87]. Copyright 2017, Advanced Materials. (**d**) Relative conductance change (ΔG/G_0_) vstime in seconds for a multilayer BP sensor showing sensitivity to NO_2_ concentrations (5–40 ppb). The inset shows a zoomed-in image of a 5 ppb NO_2_ exposure response with the identification of points in time where the NO_2_ gas is switched on and off [88]. Copyright 2015, American Chemical Society.

Graphene possesses excellent flexibility, facile surface functionalization, superior mechanical strength, and other outstanding performance characteristics, making it extensively applied in the realm of gas sensors. Kim et al. [84] have developed a flexible and transparent gas sensor based on graphene prepared by using the CVD method, as shown in Figure 8a. The sensor demonstrated reliable sensing performance even after repeated mechanical bending, as shown in Figure 8b, using NO_2_ with a mass fraction of 5 × 10^−6^ as an example.

**Figure 9:**
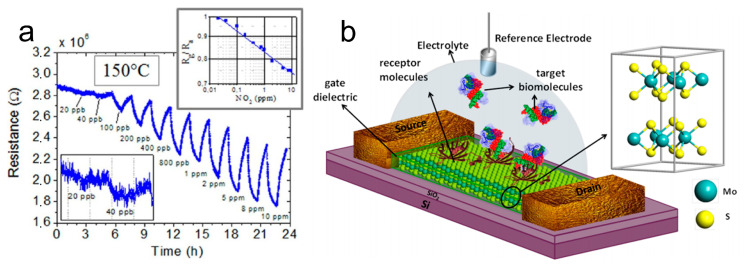
(**a**) GO response to increasing NO_2_ concentration at 150 °C. Device resistance versus time is reported in the main panel. Bottom-left inset: zoomed-in view of the detection limit range. Top-right inset: sensitivity curve [85]. Copyright 2013, American Chemical Society. (**b**) Schematic diagram of MoS_2_-based FET biosensor [93]. Copyright 2014, American Chemical Society.

In addition, functionalized graphene has found extensive applications in gas sensing. For example, Preziso et al. [85] employed a spin-coating technique to deposit large sheets of graphene oxide (GO) aqueous solution onto Pt electrodes for the fabrication of gas sensors. It has the capability of detecting NO_2_ at a mass fraction of 2 × 10^−8^ and exhibits a responsivity of 2.8% (Figure 9a). Wang et al. [86] reported that the 2D rGO/WS_2_ composite sensor demonstrated a significantly improved response to low concentrations of NH_3_ (10–50 ppm) at room temperature, surpassing that of the individual rGO and WS_2_ ones. The enhanced sensitivity can be attributed to the presence of a greater number of functional groups in rGO NSs and the introduction of additional acid centers through WS_2_ nanoflake doping. Furthermore, the sensor exhibits excellent selectivity toward potential interferents such as NO_2_, alcohols, acetone, and benzene, in addition to displaying remarkable long-term stability.

Additionally, other 2D NMs, such as MoS_2_ and black phosphorus (BP), can be used to prepare highly sensitive gas sensors by controlling the number of layers and modulating the bandgap. Zhao [87] presented an integrated array of monolayer molybdenum disulfide (ML-MoS_2_) humidity sensors with exceptionally high sensitivities for moisture mapping. As shown in Figure 8c, the resistance of the MoS_2_ field-effect transistor (FET) was 1.27 × 10^7^ Ω in dry air (relative humidity, RH = 0%). In contrast, when RH = 35% at a gate voltage (V_G_) = 30 V, the resistance increases to approximately 8.3 × 10^11^ Ω. The significant variation in resistance, exceeding 10^4^ Ω, can be attributed to the efficient adsorption of water molecules on the meticulously cleaned and patterned surface of MoS_2_. Abbas et al. [88] employed the black scale-based gas sensor to detect NO_2_ with a mass fraction as low as 5.0 × 10^−9^ and achieved a high responsivity of 2.9% (Figure 8d). In order to achieve high sensitivity to trace NO_2_ gas at room temperature (25 °C), Zhou et al. [89] selected a composite of fewer-layered MoS_2_ Nss and ZnO as the sensors. The as-prepared MoS_2_/ZnO sensors exhibited a response of 188 toward 200 ppb NO_2_, a sensitivity of 0.93/ppb, and a detection limit as low as 50 ppb. This excellent sensing performance can be attributed to the synergistic combination of ZnO and MoS_2_ NSs, which results in a hierarchical structure that facilitates gas diffusion, as well as adsorption and desorption processes.

In addition to gas sensors, there are numerous other types of sensors that can benefit from the unique properties of 2D NMs. These mainly include (1) biosensing: with their large surface areas, high conductivity, and biocompatibility, 2D NMs are highly suitable for biosensing applications such as biomarker detection, DNA sequencing, protein sensing, and point-of-care diagnostics [90]. (2) Strain and pressure sensing: due to their exceptional mechanical properties, 2D NMs can be used in strain and pressure sensing applications. Their electrical conductivity can be modulated by applied strain or pressure, enabling the development of flexible and wearable sensors [91]. (3) Chemical sensing: the applications of 2D NMs in chemical sensing have been extensively studied, including the detection of pollutants, heavy metals, and toxic substances. Their high sensitivity and rapid response make them highly promising candidates for environmental monitoring and industrial safety purposes [92]. For example, Banerje et al. [93] reported the fabrication of FET sensors for pH and biomolecule detection based on mechanically exfoliated MoS_2_ NSs as channel materials (Figure 9b). Upon changes in pH value, the FET sensor exhibited noticeable variations in current, which can be explained by the alteration in surface charge caused by the protonation/deprotonation of the OH groups on the gate dielectric. In order to detect biomolecules, the dielectric layer covering the MoS_2_ channel was modified with biotin to capture streptavidin. In response to streptavidin, the FET sensor showed a significant decrease in current due to the gating effect caused by the negative charge of streptavidin. Triboelectric nanogenerators (TENGs) are used to power devices by converting friction into electrical energy. This has important applications in the medical field. Kim et al. [94] developed a wearable electrocardiogram (ECG) system powered by a thermoelectric generator (TEG), which can generate over 13 µW cm^−2^ of power for more than 22 h through temperature differences. Nisha [95] has designed a novel sensing configuration comprising 2D NMs, Au, and magnetic Ni. The proposed sensor exhibits superior sensitivity when compared to the conventional Au film-based surface plasmon resonance (SPR) sensor. As a result, the optimized MoS_2_ and graphene layers exhibit a high sensitivity of 229°/RIU in an angular interrogation method. Hassel [96] proposed an innovative approach for detecting THz radiation through antenna-coupled mechanical resonators that rely on atomically thin 2D NMs. The sensitivity of the proposed detectors stems from the exceptional mechanical and electrical properties of atomically thin graphene or graphene-related 2D NMs.

### 4.2. Lithium-Ion Battery

**Figure 10:**
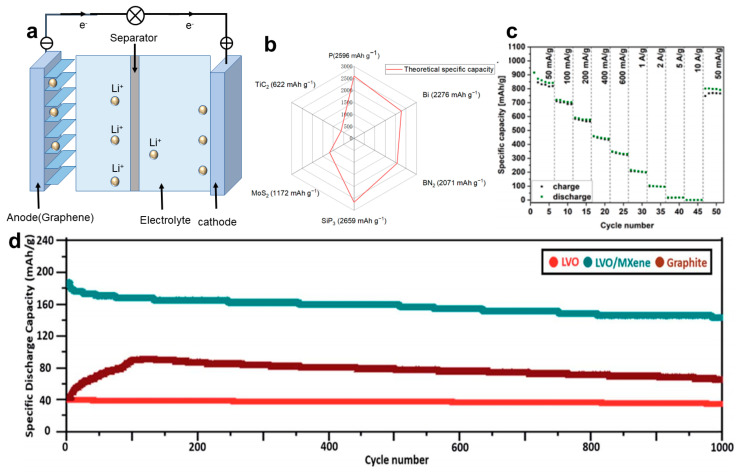
(**a**) Schematic diagram of LIB work. (**b**) Theoretical specific capacity of some 2D NMs used in LIBs. (**c**) Rate performance of ERGO/MoS_2_ films recorded at different current densities [94]. Copyright 2022, Electrochimica Acta. (**d**) Capacity retention under 5 C [95]. Copyright 2019, The Royal Society of Chemistry.

Two-dimensional NMs like MXene have garnered significant attention in the field of Li-ion batteries (LIBs) due to their shorter solid-state diffusion lengths, which can lead to improved rate performance [97]. LIBs are a kind of secondary battery that primarily rely on the movement of Li^+^ between positive and negative electrodes to function, as illustrated in Figure 10a. Therefore, the primary performance parameters of 2D NMs for application in LIBs are battery capacity (Figure 10b), cycle life, and internal resistance [98]. Given their exceptional performance characteristics, the materials can be utilized as anode materials and functional separators within such batteries.

The primary function of the anode in LIBs is to facilitate the intercalation and deintercalation of Li^+^ during charging and discharging, respectively. Due to their layered structure, two-dimensional NMs provide a favorable pathway for Li^+^ diffusion while maintaining structural stability throughout cycling. Therefore, they are promising candidates as anode materials. Syamsai et al. [99] successfully prepared bi-metallic titanium-tantalum carbide (Ti*_x_*Ta_(4−*x*)_C_3_) MXene. The Ti*_x_*Ta_(4−*x*)_C_3_ MXene exhibited a remarkably high reversible specific discharge capacity of 459 mAh g^−1^ as a Li-ion host anode, with a coulombic efficiency of approximately 99% after 200 cycles and a capacity retention of about 97%. Qian et al. [100] developed a versatile electrodeposition method for fabricating robust, flexible, and self-supporting MXene@M (M = Sb, Sn, and Bi) anodes for LIBs. Thanks to the incorporation of the 2D MXene buffer layer, these hybrid anodes demonstrated remarkable structural stability when compared to bulk metal anodes without MXene. For instance, the MXene@Sb anode consistently maintained a high reversible capacity of 516.8 mAh g^−1^ over 500 cycles.

In 2022, Kędzierski et al. [101] developed anode materials composed of self-expanding GO (ERGO) and exfoliated MoS_2_ (ex-MoS_2_) in a ratio of 1:1, named ERGO/MoS_2_. The specific capacity of this material reached 916 mAh g^−1^ at a current density of 50 mA g^−1^ and remained at 801 mAh g^−1^ after undergoing 50 cycles, as shown in Figure 10c. The Li_3_VO_4_ (LVO)/Ti_3_C_2_T_x_ composite electrode, developed by Huang et al. [102], exhibits a capacity retention of 187 mAh g^−1^ after one charge-discharge cycle at 5 C and retains a capacity dropped to 146 mAh g^−1^ after 1000 cycles. Its performance surpasses that of graphene electrodes and LVO electrodes, as illustrated in Figure 10d.

### 4.3. Photodetector

**Figure 11:**
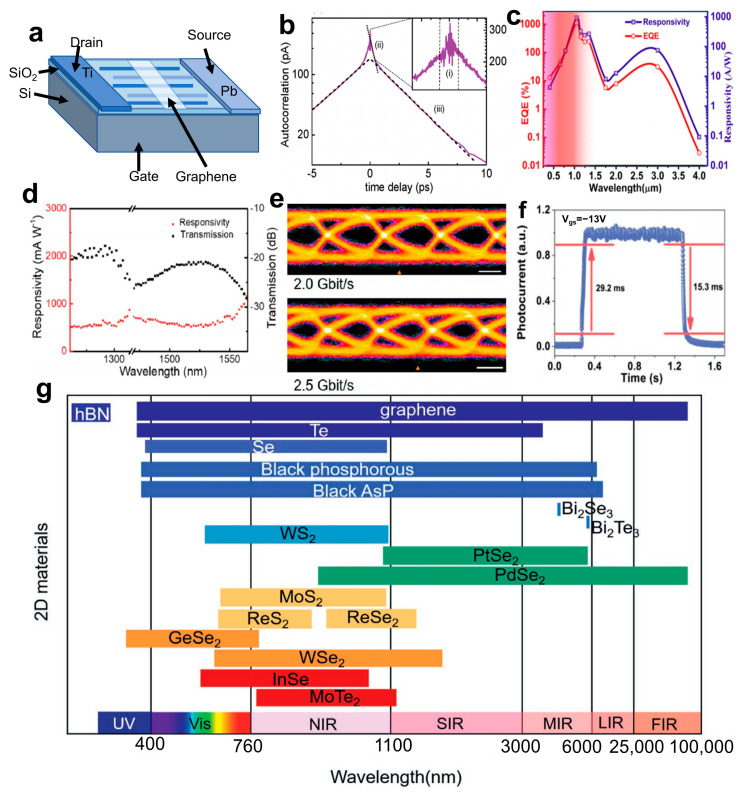
(**a**) Schematic diagram of metal-graphene-metal photodetector structure. (**b**) Photocurrent autocorrelation signal diagram. Part (i) is due to interference of the lasers pulses. Part (ii) corresponds to a subpicosecond contribution associated with carrier relaxation via phonons, part (iii) shows a contribution on a picosecond time scale connected to the response time of the photodetector. The response time is determined from (iii) by a linear fitting procedure of the right- and left-sided parts (dashed lines) of the autocorrelation function and subsequent averaging [108]. Copyright 2011, American Chemical Society. (**c**) Maximum external quantum efficiency, photosensitivity versus wavelength [109]. Copyright 2020, American Chemical Society. (**d**) Spectral response of PdSe_2_ photodetectors. (**e**) Receiver eye diagram at a data rate of 2.0 and 2.5 Gbit s^−1^, measured with a PdSe_2_ photodetector. Scale bar: 200 ps [114]. Copyright 2022, American Chemical Society. (**f**) Rise and decay curves of the PWSS-InSe device [115]. Copyright 2022, Advanced Optical Materials. (**g**) Response spectra corresponding to partial 2D materials (The lines at the bottom represent various ranges of wavelengths and other lines represent different 2D NMs) [110]. Copyright 2021, Advanced Materials.

A photodetector is a device that converts radiation from the surface of a material into an electrical signal, with its performance parameters primarily including detection wavelength and photoresponsivity. Graphene plasmonics has made rapid progress in recent years owing to graphene’s unique electrical and optical properties, tunability, long-lived collective excitation, and extreme light confinement [103]. Two-dimensional NMs, like BP and TMDs, possess several advantages, such as high surface specific area, atomic-level flatness, high carrier mobility, strong matter-light interactions, robust mechanical toughness, and effective gate control while lacking any surface dangling bonds. Additionally, they exhibit high portability [104]. Therefore, graphene and other 2D NMs, such as transition metal dichalcogenides (TMDs), have emerged as highly promising building blocks for opto-electronic applications, particularly in the field of photodetection [105]. Single-element 2D NMs are extensively employed in high-frequency, broad-spectrum photodetectors due to their excellent properties, including high mobility and a narrow band gap [106]. Polat et al. [107] presented a novel wearable device that employs graphene sensitized with semiconducting quantum dots (GQD), exhibiting exceptional tunability in responsivity and achieving a peak responsivity of approximately 10^−5^ A W^−1^ near the charge neutrality point (CNP). Moreover, their approach offers a scalable solution for integrating graphene into fully flexible wearable circuits, enhancing their physical appearance, tactile sensation, durability, and functionality. The metal-graphene-metal photodetector structure (Figure 11a) developed by Alexander et al. [108] exhibits an ultra-short intrinsic response time of 2.1 ps at a bandwidth of 262 GHz, as shown in Figure 11b. However, the photoresponsivity of graphene-based photodetectors is limited. Nonetheless, using alternative single-element 2D NMs such as arsenic (As) can address the above problems. Nidhi et al. [109] developed a photodetector utilizing a black arsenic-silicon hybrid material that achieves an ultra-wide detection range from 405 nm to 4 μm. The detector also exhibits high spectral response (>10 A W^−1^) and external quantum efficiency (>10%) across the entire optical communication band (Figure 11c). Moreover, BP has significant potential as a material for detectors operating in the visible-to-mid-infrared range due to its direct bandgap that ranges from 0.3 eV (bulk) to 2.0 eV (monolayer) [110]. A high-performance top-gated phototransistor based on BP has been achieved [111], enabling broadband detection across visible and infrared to THz wavelengths. The device demonstrated a photovoltaic effect in both visible and infrared frequencies while exhibiting a photo-thermoelectric effect at longer wavelengths. The phototransistor exhibited a responsivity range of 5 to 60 V W^−1^ and a low noise equivalent power (NEP) of less than 0.1 nW Hz^−1/2^ within the frequency range of 20 to 40 GHz.

However, single-element 2D NMs exhibit high chemical reactivity and are susceptible to oxidation in air. In contrast, dual-element 2D NMs demonstrate superior stability [112]. Therefore, more attention is being paid to investigations on photodetectors based on dual-element 2D NMs. PdSe_2_ possesses unique properties that enable it to achieve broadband detection. The bandgap of PdSe_2_ and its unique pentagonal atomic structure is dependent on the thickness, allowing for a narrowing from 1.3 eV in a single layer to 0 eV in bulk. The devices fabricated using fewer-layered PdSe_2_ have demonstrated exceptional ambipolar semiconducting characteristics, including high electron-apparent field-effect mobility at room temperature, reaching up to 158 cm^2^ V^−1^ s^−1^ [113]. Additionally, photodetectors based on PdSe_2_ were fabricated by Wu et al. [114], exhibiting a high responsivity of 1758.7 mA W^−1^ and an external quantum efficiency of 95% at a wavelength of 1550 nm, with a transmission speed exceeding 2.5 Gbit s^−1^ (Figure 11d,e). Yang et al. [115] employed WSe_2_ as a substrate passivation layer, which facilitates the separation and prolongs the lifetime of photo-generated charge carriers, resulting in enhanced photoresponsivity. The detector showed excellent performances with a photoresponsivity of 112 W A^−1^ and response/decay time of 29.1/15.3 ms (Figure 11f). The MoS_2_ [116] photodetector exhibited a positive photoresponse in the visible regime and a negative photoresponse in the infrared regime, whereas the transistor displayed a negative photocurrent due to the bolometric effect. The highest responsivity of up to 105 A W^−1^ at 454 nm was achieved through a combination of the photogate effect and the charge-trapping mechanism during positive photoresponse. In contrast, when operating in the infrared regime state, it demonstrated its highest infrared responsivity. Topological insulators have emerged as highly promising candidates for opto-electronic devices due to their rapid and dynamic electron response under optical excitation [110]. A back-gated FET utilizing Bi_2_Se_3_ flakes was successfully demonstrated [117], exhibiting remarkable responsivity with an external quantum efficiency (EQE) of 233% and a high D* (D* = $\frac{\surd A_{d}}{NEP}$) value of 3.3 × 10^10^ Jones at 300 K under 1456 nm irradiation. Notably, the device achieved an impressive responsivity of 2.7 A W^−1^. The response spectra corresponding to partial 2D NMs is shown in Figure 11g.

### 4.4. Electromagnetic Wave Absorption

Two-dimensional NMs exhibit exceptional electromagnetic shielding and microwave absorption properties owing to their large specific surface area that facilitates the penetration and attenuation of electromagnetic waves. As a result, they can significantly enhance effective absorption bandwidth and reflection loss. Additionally, 2D NMs are capable of altering their bandgap through layer manipulation [118], rendering them a promising candidate for electromagnetic wave absorption applications and protective measures in electronic equipment, communication systems, wireless identification technologies, and other applications.

**Figure 12:**
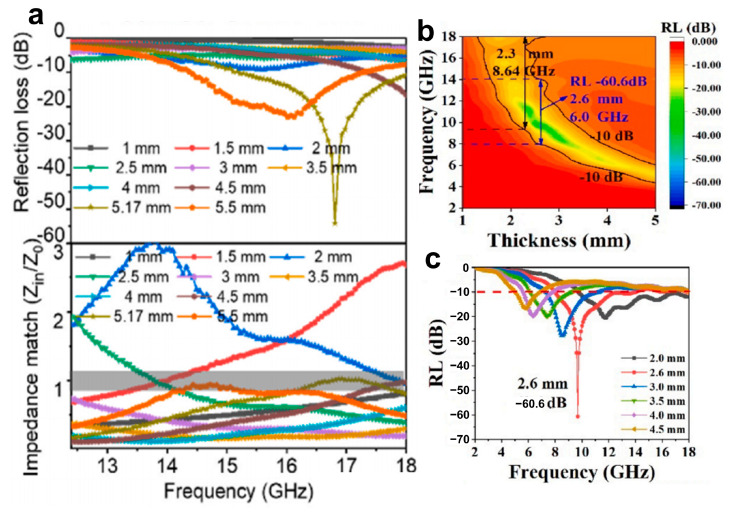
(**a**) Impedance loss and impedance matching of graphene with different thicknesses [119]. Copyright 2022, Carbon. (**b**) Effective absorption bandwidth and reflection loss of MXene nanosheets. (**c**) Two-dimensional RL curves of MXene nanosheets [120]. Copyright 2023, Carbon.

Recently, Ma et al. [119] have developed a graphene-based material for electromagnetic wave absorption that exhibits improved absorption properties and effective impedance matching. As shown in Figure 12a, the material achieves a reflection loss of −54.1 db at 16.8 GHz. Yv et al. [120] utilized polyaniline nanorods for the in situ modification of MXene NSs, resulting in an effective absorption bandwidth of 8.64 GHz at a material thickness of 2.3 mm (Figure 12b) and a reflection loss of −60.6 db at a material thickness of 2.6 mm (Figure 12c).

### 4.5. Photocatalysis

**Figure 13:**
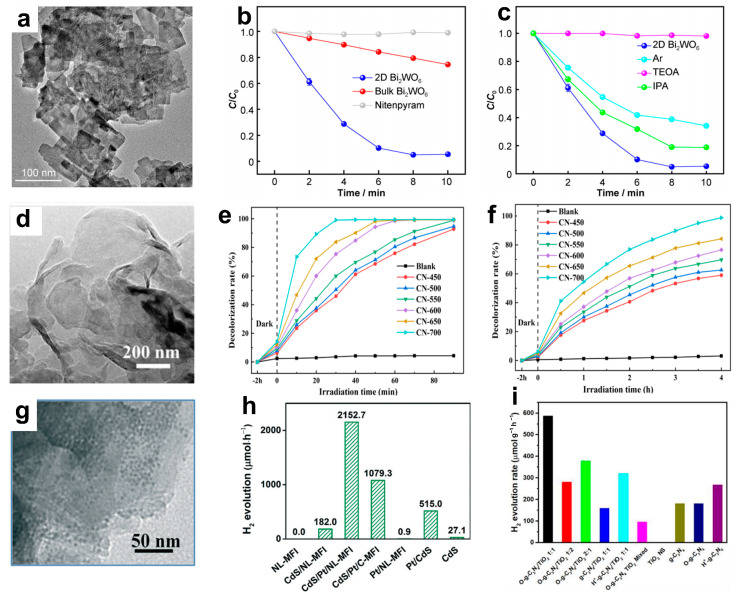
(**a**) TEM image of 2D Bi_2_WO_6_. (**b**) Degradation curves of NTP over different samples. (**c**) Degradation curves of NTP under different scavengers for 2D Bi_2_WO_6_ [122]. Copyright 2022, Rare Metals. (**d**) TEM image of CN-700 (**e**) Photocatalytic decolorization rate curve of g-C_3_N_4_ (RhB). (**f**) Photocatalytic decolorization rate curve of g-C_3_N_4_ (MO) [123]. Copyright 2022, Catalysts. Enhancement of Pt-based catalysts.) (**g**) TEM image of CdS/Pt/NL-MFI. (**h**) H_2_ evolution rates on different photocatalysts [124]. Copyright 2019, Catalysis Science & Technology. (**i**) Hydrogen evolution rates of all tested photocatalysts [125]. Copyright 2017, Applied Catalysis B: Environmental.

As environmental pollution and the energy crisis worsen, semiconductor photocatalysis—which includes photodegradation pollutants, water splitting, CO_2_ reduction, and others—has become an area of intense research. Solar-light-driven photocatalysis involves processes of charge carrier generation, separation/transfer, and surface reaction. Due to their distinctive layered structure, in-plane anisotropy, ultra-high carrier mobility, and tunable bandgap, 2D NMs, like C_3_N_4_ and TMDs, have tremendous potential for use in photocatalysis [121]. Nitenpyram (NTP), being a widely used insecticide, presents significant risks to both human health and environmental safety. In order to address this issue, Cheng et al. [122] synthesized a visible-light-responsive photocatalyst of two-dimensional Bi_2_WO_6_ (Figure 13a) and applied it to the degradation of NTP. As shown in Figure 13b, no degradation of NTP was observed under visible-light irradiation in the absence of a photocatalyst. When using 2D Bi_2_WO_6_ and bulk Bi_2_WO_6_ as photocatalysts, the degradation percentages of NTP were observed to be 94.6% and 25.4%, respectively. Obviously, 2D Bi_2_WO_6_ exhibits more highly enhanced activity than bulk Bi_2_WO_6_, which can be attributed to its unique 2D structure that promotes the separation of photo-generated charge carriers. Furthermore, the reactive species trappin experiments show that photo-generated h^+^ and •O^2−^ are the main active species for 2D Bi_2_WO_6_ in NTP degradation (Figure 13c). Ke et al. [123] found that the photocatalytic performance of g-C_3_N_4_ was influenced by different calcination temperatures, and the sample calcined at 700 °C (Figure 13d) exhibited the highest activity for degrading rhodamine B (RhB) and methyl orange (MO), achieving degradation rates of 99.11% and 98.81%, respectively (Figure 13e,f). Additionally, the optimized g-C_3_N_4_ exhibits good reusability. The graphene-like structure enables a large surface area, efficient light harvesting ability, and the boosted separation and transfer of photo-generated charge carriers, thereby improving photocatalytic performance.

Hydrogen (H_2_) is the ideal replacement for fossil fuels due to its renewability, safety, high energy density, and environmental friendliness. Photocatalytic H_2_ generation from water splitting is a promising strategy for solving energy and environmental crises. Therefore, the development of efficient H_2_ evolution catalysts based on 2D NMs is of great significance. Liu et al. [124] fabricated a porous lantern-like MFI (NL-MFI) zeolite composed of 2D NSs, which was then utilized as a support for Pt and CdS nanoparticles to form a highly efficient CdS/Pt/NL-MFI photocatalyst (Figure 13g). As expected, the CdS/Pt/NL-MFI photocatalyst demonstrated a remarkable H_2_ evolution rate of 2152.7 μmol h^−1^ under visible light irradiation (Figure 13h), with an apparent quantum efficiency of 39.4%. This signifies a significant improvement in the rate of H_2_ evolution compared to that achieved using CdS and Pt nanoparticles supported by commercial MFI zeolite (1079.3 μmol h^−1^). The NL MFI zeolite support enhances visible light absorption, facilitates photo-generated electron-hole pairs separation, and promotes water molecule interactions with the photocatalyst.

Zhong et al. [125] utilized an in situ solvothermal synthetic strategy to construct a covalently bonded oxidized graphitic C_3_N_4_/TiO_2_ (2D/2D O-g-C_3_N_4_/TiO_2_) heterostructure with a high surface area. The resulting hybrid exhibited a remarkable 6.1-fold enhancement in visible-light photocatalytic activity for H_2_ evolution (587.1 μmol h^−^^1^ g^−^^1^) when compared to a physical mixture of TiO_2_ NSs and 3.2 times higher than that of O-g-C_3_N_4_ (Figure 13i). This can be attributed to the formation of a heterojunction via interfacial N-O-Ti bonding, which facilitates the migration of charge carriers. Additionally, 2D TMDs have been extensively studied as cocatalysts for H_2_ evolution. For example, the incorporation of 2D MoSe_2_ could significantly enhance the efficiency of visible-light-driven H_2_ generation on CdS [126].

**Figure 14:**
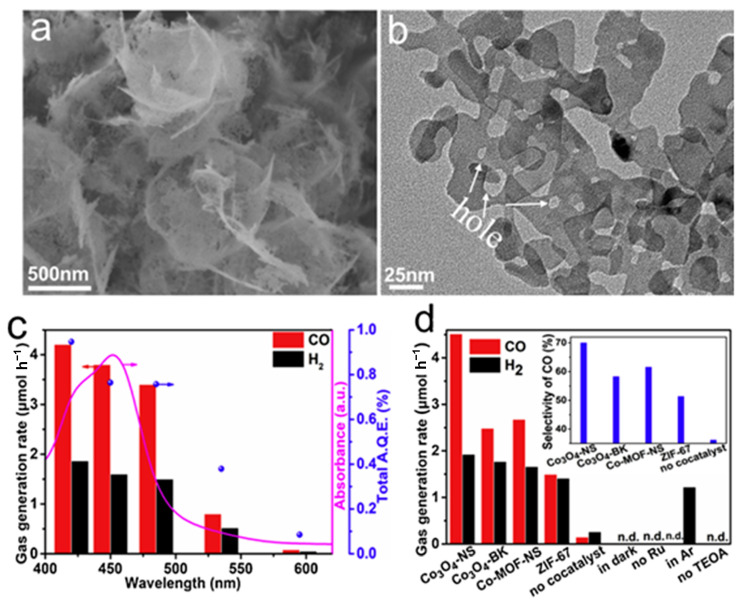
(**a**) SEM image and (**b**) TEM image of Co_3_O_4_-NS. (**c**) Wavelength dependent on CO and H_2_ generation. The line is the absorption spectrum of the Ru photosensitizer. The dots are the total AQE value for each wavelength. (**d**) Evolution of CO and H_2_ under various reaction conditions. The inset shows CO selectivity [128]. Copyright 2018, Applied Catalysis B: Environmental.

The chemical reduction of CO_2_ to hydrocarbons plays a crucial role in the Earth’s carbon cycle and holds immense potential as a future technology for capturing and storing solar energy. Two-dimensional photocatalysts usually show enhanced photocatalytic activities that can be attributed to the short diffusion length of electrons and holes to the surface, as well as the highly accessible active sites [127]. Chen et al. [128] developed a facile, scalable, and controllable approach to preparing ultrathin 2D porous Co_3_O_4_ catalysts (Co_3_O_4_-NS, Figure 14a,b) by air calcining ultrathin metal-organic framework (MOF) nanosheet templates, which had excellent stability in CO_2_ reduction. The resulting Co_3_O_4_-NS catalysts were effectively utilized for CO_2_ reduction with a Ru-based photosensitizer under visible light irradiation, resulting in a CO generation rate of approximately 4.52 μmol h^−^^1^ with a selectivity of 70.1%, surpassing that of Co_3_O_4_ bulk catalysts (Co_3_O_4_-BK) (Figure 14c,d). Co_3_O_4_-NS inherited s 2D morphology and well-developed porosity from the MOF precursors, which facilitated electron transport, enhanced CO_2_ molecule adsorption, and provided abundant catalytic sites for CO_2_ activation.

### 4.6. Electrocatalysis

**Figure 15:**
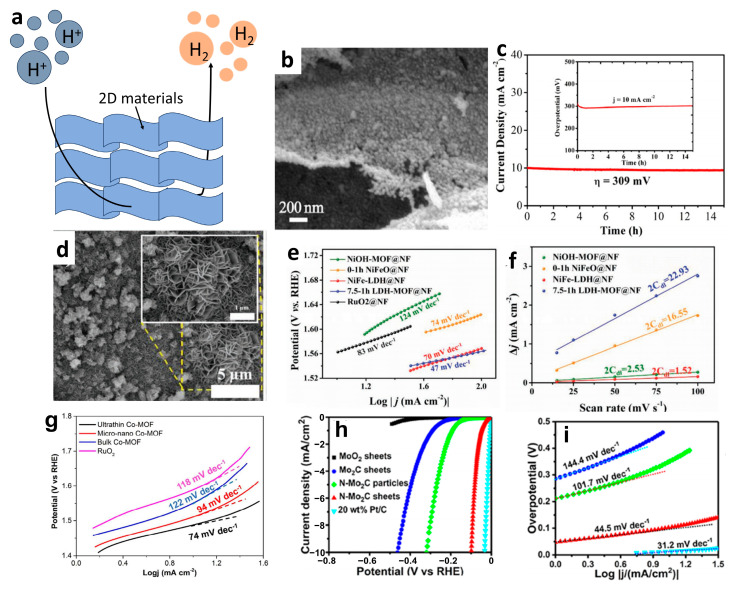
(**a**) Diagram of electrocatalytic hydrogen evolution mechanism. (**b**) SEM images of Ni_3_C/NC nanoflakes. (**c**) Chronoamperometry curve at an overpotential of 309 mV (the inset is chrono-potentiometric curve at current density of 10 mA cm^−^^2^) [131]. Copyright 2019, Electrochimica Acta. (**d**) SEM images of pristine 7.5–1 h LDH-MOF@NF. (**e**) Corresponding Tafel slopes in 1 M KOH. (**f**) *C*_dl_ values of different samples in 1 M KOH for comparison [132]. Copyright 2019 WILEY-VCH Verlag GmbH & Co., KGaA, Weinheim. (**g**) Tafel slopes of ultrathin 2D Co-MOF nanosheets, micro-nano Co-MOFs, bulk Co-MOFs and RuO_2_ [134]. Copyright 2018, Journal of Materials Chemistry A. (**h**) Polarization curves of MoO_2_ NSs, Mo_2_C NSs, N-Mo_2_C nanoparticles, N-Mo_2_C NSs, and 20 wt% Pt/C on the GC electrode at 5 mV s^−1^ in 0.5 M H_2_SO_4_; (**i**) corresponding Tafel slopes from (**h**) [135]. Copyright 2017, ACS Nano.

Energy is poised to become the core resource of competition among countries in the future, and electrocatalysis will play a crucial role in energy generation, transportation, and consumption (Figure 15a). Therefore, it is imperative to promote the advancement of electrocatalysis. It is well known that some 2D NMs, such as MOFs NSs and MXene, have extensive applications in electrocatalysis. The potential of 2D NMs in catalysis is attributed to their large surface area, exceptional mechanical properties, and high thermal and electrical conductivity. The high specific surface area of 2D NMs provides abundant active sites, while their superior mechanical properties ensure catalytic durability [129]. Additionally, thermal conductivity facilitates efficient thermal diffusion during reactions. Moreover, the tunable electronic properties of 2D NMs enable control over catalytic performance, resulting in superior catalytic stability and activity compared to bulk materials [130].

For example, Zheng et al. [131] fabricated Ni_3_C/N-doped carbon (NC) nanoflakes with a controlled structure and composition through the adjustment of the synthetic conditions (Figure 15b). The resulting Ni_3_C/NC nanoflakes exhibited remarkable electrocatalytic performance towards OER, achieving a low overpotential of 309 mV, as shown in Figure 15c. Additionally, Chen et al. [132] synthesized NiFe-based MOF nanosheet arrays (LDH-MOF NS) (Figure 15d) derived from layered double hydroxides (LDH), which exhibited superior electrochemical oxygen evolution reaction (OER) performance with a low Tafel slope of 47 mV dec^−^^1^ (Figure 15e), surpassing the benchmark RuO_2_ and most of the state-of-the-art catalysts (Figure 15f). The bimetallic 2D MOF-Fe/Co(1:2) NSs developed by Ge [133] exhibited excellent electrocatalytic activity towards OER in alkaline solutions, characterized by an overpotential of 238 mV at 10 mA cm^−^^2^, which is much lower than those of bulk materials and 3D MOF-Fe/Co(1:2). The superior OER activity can be attributed to the high electrochemical active surface area. The highly efficient ultrathin 2D Co-MOF NSs towards OER in alkaline solutions were reported by Xv et al. [134], surpassing the performance of most Co_3_O_4_ and MOF-based electrocatalysts (Figure 15g). Jia et al. [135] fabricated N-Mo_2_C NSs via a two-step approach and reported their remarkable HER activity, characterized by a low overpotential (99 mV versus RHE at 10 mA cm^−2^) and Tafel slope (44.5 mV dec^−1^), as shown in Figure 15h,i. Zang et al. [136] showed that the incorporation of transition metal ions (Mo5+, W6+, and Co2+) into gelatin-based precursors could effectively promote the formation of MXene at a relatively low temperature of 600 °C. The proposed method facilitates the one-step synthesis of binary/ternary carbides/hybrids, such as Mo_2_C-Co, Mo_2−x_W_x_C, and others. HER testing revealed that Mo_2_C-Co demonstrated the most favorable performance, characterized by a Tafel slope of 39 mV dec^−^^1^ and the lowest overpotential of 48 mV at a current density of 10 mA cm^−^^2^. Transition metal nitrides (TMNs) are considered a promising class of materials for OER due to their metallic properties, which enhance electrical conductivity and reduce internal potential loss. As reported by Xu et al. [137], Ni_3_N NSs with a thickness of 3 nm exhibited excellent OER activity with a small Tafel slope of 45 mV dec^−^^1^ in an O_2_^−^ saturated basic solution (1 M KOH).

## 5. Summary

**Figure 16:**
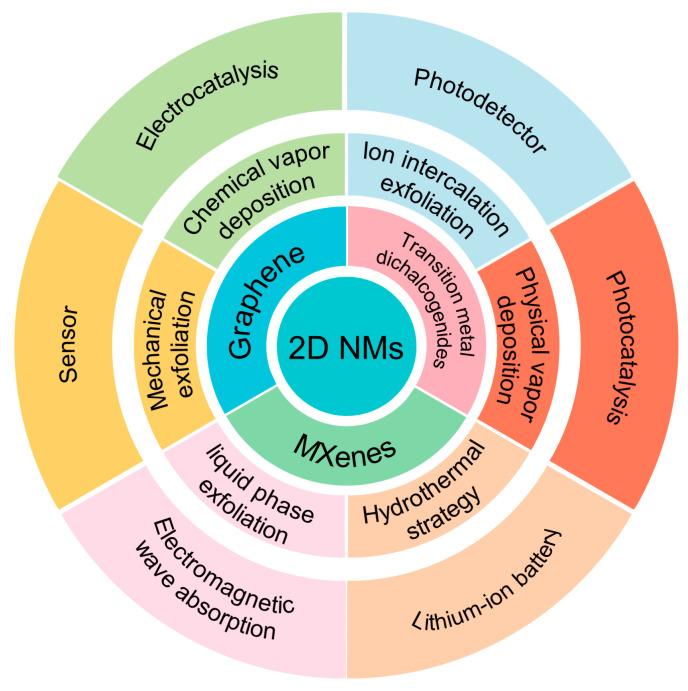
Preparation and applications of typical 2D NMs.

This paper examines the characteristics of various methods for preparing 2D NMs and reviews the recent research progress in this field. The different preparation techniques are compared, and the strategies for optimizing the preparation process are summarized. Additionally, this paper discusses the current applications and future development directions for 2D NMs. Figure 16 summarizes the preparation and applications of some 2D NMs.

### 5.1. Preparation

The mechanical exfoliation method boasts low costs and avoids the corresponding chemical changes during preparation, resulting in higher sample purity. However, it also presents some issues, such as a low yield and an uneven sample thickness. By altering the medium or incorporating adhesives, the current mechanical peeling technique can enhance production efficiency. Additionally, pollution-free high-quality 2D NMs can be produced by modifying the stripping environment to ultra-vacuum conditions. The ultrasonic liquid-phase-assisted exfoliation method primarily affects the morphology and size of 2D NMs by altering the solvents. Therefore, selecting an appropriate solvent can achieve the desired effect. However, this method still faces challenges, such as limited and small sample size and low yield. Currently, ultrasonic liquid-phase-assisted exfoliation is advancing towards utilizing multiple solvents in a synergistic manner.

The ion intercalation stripping method exhibits a high yield for 2D NMs NSs; however, the regulation of the amount of ion intercalation remains challenging. Currently, lithium ions are predominantly employed for intercalation. The CVD method offers the advantage of superior manipulation and pollution avoidance during preparation, making it significantly more advantageous than the “top-down” method. The resulting product has a wide range of applications. However, this method is faced with certain disadvantages, such as high costs, raw material volatility, and low reactant utilization rates. Currently, the regulation of this product is primarily dependent on adjusting various conditions, such as temperature, pressure, and distance, in order to ensure optimal performance. The hydrothermal method is characterized by its simplicity of operation, low cost, process stability, and high product quality. However, it still suffers from a prolonged reaction cycle and is generally limited to the preparation of oxide products. The hydrothermal method primarily controls the morphology of products by altering the solvent composition or pH value in the reactor, as well as adjusting the filling degree and other conditions.

### 5.2. Application

Some 2D NMs, like graphene, TMDs, etc., exhibit great potential for gas sensing applications by virtue of their good gas adsorption, large specific surface area, excellent electrical conductivity, and stable chemical properties. Moreover, sensors based on 2D NMs can detect gases with mass fractions as low as 10^−9^ orders of magnitude while maintaining high selectivity towards target gases. Additionally, 2D NMs have attracted significant attention in the field of LIBs due to their rapid charge carrier transport, ion diffusion pathways, and synergistic interactions between carriers and ions. LIBs based on 2D NMs exhibit a prolonged cycle life and exceptional capacity retention. Further, the utilization of 2D NMs as photodetectors can provide high sensitivity and a short response time due to their unique properties, including a high surface area-to-volume ratio, atomic-level flatness, high carrier mobility, strong matter–light interactions, robust mechanical toughness, and gate controllability. Additionally, these materials are highly portable and do not possess any dangling bonds on their surfaces. Traditional absorbing materials exhibit limited reflection loss and a narrow absorption range. In contrast, 2D NMs possess exceptional electromagnetic shielding and microwave absorption properties, as well as a large specific surface area that facilitates the penetration and attenuation of electromagnetic waves. As such, incorporating 2D NMs can significantly enhance the performance of absorbing materials. Besides, the unique layered structure, in-plane anisotropy, ultra-high carrier mobility, and adjustable bandgap of 2D NMs endow them with vast potential for photocatalysis applications, including, but not limited to, the photodegradation of pollutants, water splitting, and CO_2_ reduction. Further, 2D NMs are crucial in the field of electrocatalysis due to their large surface area, exceptional mechanical properties, and high thermal and electrical conductivity.

## 6. Prospect

Substantial advancements have been achieved in the research on the preparation and application of 2D NMs. The mechanical exfoliation method is widely utilized for laboratory research and device fabrication to prepare 2D NMs. Moreover, CVD can produce large-area, high-quality graphene and some TMDs, which provides a solid foundation for the commercialization of 2D NMs. Secondly, with regards to application, the gas sensor is developed based on the robust adsorption capacity of 2D NMs toward gases and their high sensitivity toward environmental fluctuations. Thanks to their capacity for responding to visible light and excellent chemical stability, 2D NMs have found widespread use in the field of photocatalysis, demonstrating their great potential for preparation and application.

The research on 2D NMs still faces numerous challenges, particularly in terms of preparation, where the current level is insufficient for producing high-end devices. Although mechanical exfoliation is a commonly used method for preparing 2D NMs, it suffers from low efficiency and uncontrollable sample thickness. Other preparation methods, such as CVD, have a broad range of sample applications; however, they are associated with high costs and harsh preparation conditions. In the future, the development trends of 2D NMs may encompass the following aspects: (1) the exploration and investigation of novel 2D NMs. As research advances, there is a possibility of discovering new 2D NMs. The exploration and study of these emerging materials will contribute to expanding the application field and enhancing the performance characteristics of 2D NMs. (2) Research on multifunctional 2D NMs. The unique structure and properties of 2D NMs endow them with a wide range of potential applications, including electronic devices, sensors, catalysts, and other fields. In the future, the focus of research on 2D NMs will increasingly revolve around their development and versatile application. (3) The improvement and advancement of preparation technology for 2D NMs. The preparation technology of 2D NMs plays a crucial role in determining their performance and application potential. In the future, enhancing and developing the preparation technology will lead to more efficient, controllable, and large-scale production of 2D NMs. (4) The expansion of the application scope of 2D NMs. With the advancement of research on 2D NMs, their applications are poised to expand further, particularly in domains like energy and biomedicine. In the future, the prospects for utilizing 2D NMs will be broader and will exert a profound influence on societal development. Finally, despite the numerous reports on the advanced devices and excellent performance of 2D NMs in various applications, there remains an urgent need to develop novel application devices, optimize existing device performance, expand application fields, and fully exploit the unique advantages of 2D NMs.

Currently, theoretical research on 2D NMs focuses on band gap regulation, surface properties, crystal defects, and heterostructures. Band gap regulation is particularly crucial because it affects the electrical properties of materials. The band gap of 2D NMs can be effectively controlled through methods such as strain, chemical modification, doping, and heterostructures, enabling the design and optimization of materials. Research on surface properties is also of considerable importance since a better understanding of the chemical reactions, adsorption, and catalysis of materials can improve their performance. Researchers have recently discovered the surface properties and reaction mechanisms of many 2D NMs, providing new ideas and directions for their applications. The study of crystal defects and heterostructures is another hot topic in 2D NMs research. The presence of crystal defects can significantly impact the mechanical, optical, and electrical properties, whereas heterostructures have the potential to introduce novel functionalities and properties. Therefore, studying the crystal defects and heterostructure of 2D NMs is essential for optimizing their performance and functional design. In summary, the theoretical development of 2D NMs is an ongoing process of exploration and discovery. Future research will be dedicated to optimizing performance and functional design to meet the diverse application requirements of various fields.

## Figures and Tables

**Table 1:** Advantages and disadvantages of various methods for the preparation of 2D MNs.

Method		Advantage	Disadvantage
Top-down method	Mechanical peeling	High sample purity, cost-effective	Low yield, poor controllability
	Ultrasonic-assisted liquid phase exfoliation	Altering the solvents affects the morphology and size, simple process	small sample size, Low yield, structural damage
	Ion intercalation-assisted exfoliation	High yield, high quality,	Poor controllability, structural damage,
Bottom-up method	Chemical vapor deposition	Pollution avoidance, excellent controllability	High costs, low reactant utilization rates
	Physical vapor deposition	High purity, uniform film deposition, precise control	Limitations in material selection, high vacuum environment, relatively slow growth rates
	Hydrothermal strategy	Low cost, process stability	Prolonged reaction cycle, impure sample
	Atomic layer deposition	Precise control, uniformity, versatility	Slower growth rate, equipment complexity, restricted reaction condition

## Data Availability

The data presented in this study are available on request.
